# Productivity and Thermal Performance Enhancements of Hollow Fiber Water Gap Membrane Distillation Modules Using Helical Fiber Configuration: 3D Computational Fluid Dynamics Modeling

**DOI:** 10.3390/membranes13100843

**Published:** 2023-10-22

**Authors:** Mohamed O. Elbessomy, Mahmoud B. Elsheniti, Samy M. Elsherbiny, Ahmed Rezk, Osama A. Elsamni

**Affiliations:** 1 Mechanical Engineering Department, Faculty of Engineering, Alexandria University, El-Chatby, Alexandria 21544, Egypt; mohamed.elbessomy@alexu.edu.eg (M.O.E.); samy.elsherbiny@alexu.edu.eg (S.M.E.); elsamni@alexu.edu.eg (O.A.E.); 2 Mechanical Engineering Department, College of Engineering, King Saud University, Riyadh 11451, Saudi Arabia; 3 Energy and Bioproducts Research Institute (EBRI), College of Engineering and Physical Science, Aston University, Birmingham B4 7ET, UK; a.rezk@aston.ac.uk

**Keywords:** thermal desalination, membrane distillation, hollow fiber MD, compact design

## Abstract

Although hollow fiber water gap membrane distillation (HF-WGMD) units offer certain advantages over other MD desalination systems, they still require enhancements in terms of distillate flux and productivity. Therefore, this work proposes a novel configuration by incorporating the helical turns of HF membranes within the water gap channel of the HF-WGMD modules. A fully coupled 3D CFD model is developed and validated to simulate the multifaceted energy conservations and diffusion mechanisms that are inherent to the transport phenomena in the proposed HF-WGMD module. Single and double helical HF membrane designs with different numbers of turns are compared to the reference modules of single and double straight HF membrane designs under various operational conditions. At a feed temperature of 70 °C, a noteworthy 11.4% enhancement in the distillate flux is observed when employing 20 helical turns, compared to the single straight HF membrane module. Furthermore, the specific productivity revealed a maximum enhancement of 46.2% when using 50 helical turns. The thermal performance of the proposed HF-WGMD module shows higher energy savings of up to 35% in specific thermal energy consumption for a one-stage module. Using three stages of single helical modules can increase the gain output ratio from 0.17 for the single stage to 0.37, which represents an increase of 117.6%. These findings indicate the high potential of the proposed approach in advancing the performance of HF-WGMD systems.

## 1. Introduction

Membrane distillation (MD) is a thermally driven technique that desalinates saline water at temperatures below the atmospheric boiling temperature. MD depends mainly on the partial vapor pressure difference across a hydrophobic membrane, which can be considered as the driving force for the process of vapor molecule diffusion. MD has different configurations depending on the method used to initiate the vapor pressure difference across membrane pores as follows: direct contact MD (DCMD) [1,2,3], in which the hot feed and cold permeate (distillate water) channels are directly in contact with the membrane surfaces that force the vapor molecules to diffuse through the membrane pores and condense in the permeate; air gap MD (AGMD) [4,5,6], in which the permeate channel is replaced by an air gap and the vapor condenses on a cold surface; sweeping gas MD (SGMD) [7,8,9], in which a relatively dry air stream is forced through the permeate channel to carry the diffused vapor molecules to be condensed outside of the desalination unit; vacuum MD (VMD) [10,11], in which a vacuum pump is applied to the permeate channel to keep a low pressure at the permeate side; and finally, water gap MD (WGMD) [12,13], in which stagnant water is in direct contact with the membrane’s cold side, and the stagnant water (water gap) is cooled from the other side using a coolant stream separated by a barrier. In WGMD, the coolant channel is separated from the water gap so that saline water can be used as a coolant to recover a part of the thermal energy that was released in the water gap. Hence, the WGMD configuration merges the features of the DCMD and AGMD techniques. In addition, WGMD performs better than the DCMD technique in terms of energy efficiency due to the thermal resistance induced by the stagnant water in the permeate side [14]. Nevertheless, it can be considered that DCMD produces a higher flux than WGMD, and both are higher than that produced by AGMD.

The membranes used in the above technologies are in the forms of sheets, tubes, or hollow fibers. Among these, hollow fiber has the most benefits because it provides a high packing density with a large surface area. Moreover, it shows good mechanical properties, easiness for assembly in bundles, and reliability in cleaning [15]. Many studies have emerged to explore the applicability of HF in the above-mentioned membrane distillation arrangements. Cheng et al. [16] conducted experiments to compare the performance of hollow fiber (HF) WGMD and HF-AGMD modules. They found that the WGMD module outperformed the AGMD module in terms of the output flux by around 8%. Also, Im et al. [17] performed experiments on an HF desalination system that consisted of a number of hollow fiber membranes and a number of hollow fibers used as permeate condensers, and all were inserted inside a polypropylene (PP) shell. The gap between the HF membranes and HF condensers was filled once with distillate water and another time with air to compare the WGMD and AGMD system performances. They observed that 27 L/(m^2^ h) of water flux was produced by the WGMD system, while only 24 L/(m^2^ h) of water flux was produced by the AGMD system, with around 12.5% of outperformance for WGMD at 80/20 °C feed/coolant inlet temperatures and tap water salinity levels. Gao et al. [14] derived experimental work on an HF-WGMD module. The module consisted of eight fibers inserted inside eight cooling tubes, all inside a shell with a 25 mm diameter and a 425 mm length. The authors used water at a 10,000 ppm salinity level for the feed and coolant channels to provide a recovery system for the evaporation’s latent heat from the feed side. The module produced water fluxes of up to 9.14 L/(m^2^ h) at 70/20 °C feed/coolant inlet temperatures while the amount of thermal energy consumed was around 6 kWh per unit kg of produced water at the same feed/coolant inlet conditions. On the other hand, they tested the same module with the same operating conditions but in the form of the HF-DCMD module. The HF-DCMD module achieved superiority in terms of flux, yet the HF-WGMD system outperformed HF-DCMD system in terms of energy efficiency by around 14%. This emphasizes the requirement to boost the HF-WGMD module’s flux.

On the other hand, experimentally validated mathematical models and computational fluid dynamics (CFD) simulations helped in investigating the different MD processes with less effort and fewer costs needed in the experimental setups. A tremendous amount of numerical and CFD models were recently built up to simulate the heat and mass transfer processes in all MD configurations with different module designs [18,19,20]. Yu et al. [21] developed a two-dimensional heat transfer model by coupling the latent heat due to evaporation into an energy conservation equation in combination with Navier–Stokes equations. The model was used to predict mass and heat transfer parameters such as the feed and permeate outlet temperatures, local heat transfer coefficients, local mass flux, and thermal efficiency. El kadi et al. [22] suggested a 2D CFD model to study the performance of a spacer-filled DCMD module and compared it with a spacer-free module at the same operating conditions. They modeled a conductive mesh of spacers on both surfaces of the MD membrane and simulated the impacts of spacers on the heat and mass transfer processes. The computational results confirmed the capability of the spacers to reduce the concentration polarization, which enhanced the mass flux by 35% compared with the mass flux produced by the spacer free module. Elsheniti et al. [8] introduced a 2D CFD model to study the effect of inserting wires, acting as turbulators, in the sweeping air channel of an SGMD module. The model had the ability to predict the local velocity, salinity, and temperature inside the three simulated domains (feed, membrane, and air channel). In addition, the model proved the effectiveness of air channel turbulators in terms of the water output flux. The authors observed 13.85 L/(m^2^ h) and 4.97 L/(m^2^ h) of distillate flux that represented 34.3% and 39% more water flux enhancements compared to that of the no-wire module at 60 and 40 °C feed inlet temperatures and seawater salinity, respectively. Additionally, some 3D CFD models of hollow fiber MD modules were introduced for DCMD and VMD configurations [23,24,25]. Zhang et al. [24] introduced a 3D CFD model to mimic the aquatic NaCl solution in the HF-VMD process. The CFD study included the effects of the membrane thickness, feed temperature, and pressure on the boundary layer development over membrane surfaces. It was observed that the majority of changes occurred on the boundary layer in the membrane silk. Yang et al. [25] provided an optimization study on the HF-DCMD desalination module. They introduced a 3D CFD study aimed at exploring the ability of HF designs to enhance the DCMD system’s performance. Hollow fibers with different geometries were included in the study such as wavy and gear-shaped cross sections to compare its performance with the original straight fibers at the same operating conditions. The authors observed a 66% enhancement in terms of the mass flux for the gear-shaped designs over the original straight fibers, followed by the wavy designs. Additionally, the CFD study included the results of the module’s hydraulic energy consumption, where the gear-shaped designs achieved the highest productivity and the lowest hydraulic energy consumption, followed by the wavy designs.

On the other hand, for the WGMD configurations, detailed numerical modeling and CFD simulations were rarely taken into account in previous studies [17,26,27,28], specifically for the HF-WGMD pattern. Specifically, Memon et al. [27] established a simple 1D numerical model to investigate the performance of a flat sheet WGMD module and compare its performance with different material gap (MG) MD modules. The modeling was divided into two parts; the first part only comprised the membrane layer mass transport model with no mass transport modeling for the other module domains, and the second one was a heat transport model for the whole MD module. The simulated output flux was increased from 2.5 to 18.4 L/(m^2^ h) when the feed inlet temperature was varied from 40 to 70 °C with around −21.9% and 1.7% deviations from the experimental fluxes, respectively. The model predicted that the water flux from the graphite-filled MGMD module was 11% to 22% higher than that of the WGMD module, while other MGMD modules such as the silica gel and zeolite MGMD modules had 17% to 24% and 18% to 27% water output fluxes, respectively, which were lower than that of the WGMD module, and they all had a 40 to 70 °C feed inlet temperature range.

Additionally, a numerical 1D model was developed by Gao et al. [28]. The authors established mass and heat transfer modeling for all module domains depending on the basic conservation laws combined with some empirical heat transfer equations. The model was used to predict the water output flux of different HF-WGMD modules such as MD modules with a single straight fiber, double straight fibers, and triple straight fibers inserted inside the module cooling tube. The module thermal energy consumption was not reported; in addition, the temperature and velocity profiles were not available with such model. Elbessomy et al. [13] developed a 2D CFD model to simulate the performance of HF-WGMD units powered by ultra-low-waste heat sources. The CFD model was used to optimize the dimensions and operating conditions of the MD module to produce the highest amount of distillate water with the minimum desalination system volume. The most compact HF-WGMD module was proposed in this study, with specifications of 91 packed fibers inside a 5 cm module shell diameter and a 10 cm shell length. The best design was found to produce water with a rate of 4.8 m^3^/day in the case of using 25/5 °C feed/coolant inlet temperatures, while the product rate could be enhanced to 12.1 m^3^/day for 45/25 °C feed/coolant inlet temperatures; both rates are per cubic meter of desalination unit.

Maintaining perfectly straight hollow fibers within the modules might seem ideal in practice. Hollow fibers typically exhibit a certain degree of waviness and mild bends, resulting in multiple potential pathways within the module vessel. This complexity calls for an exploration of alternative hollow fiber configurations that align more closely with real existing scenarios while being assembled in vessels.

From this perspective, the present study introduces a novel approach involving a mild helical configuration of hollow fibers, aiming to increase productivity and reduce the energy consumption of HF-WGMD modules. Moreover, the proposed configuration can help to release the tension stress that arises when keeping the hollow fibers straight and permit the usage of a longer length. For this purpose, a 3D CFD model that can simulate the performance of the new various configurations of helically shaped HF-WGMD models was developed in this study. The 3D model’s results and previously published experimental work were compared as part of the model validation process, and the results revealed excellent agreement.

The strategy followed in the present study was to use the CFD model to simulate various HF-WGMD units such as single and double helical fibers inserted in the module cooling tube. Both cases will be compared with the straight fiber modules (that are considered as basic references) in terms of the module productivity, thermal energy consumption, and percentage of thermal energy recovery. The geometrical and operating conditions investigated in this study include the feed temperature levels, feed and coolant inlet velocities, and the number of helical turns per module length. Carrying out this parametric study side by side with the temperature, concentration, and velocity visualizations allows for a better exploration of the flow behavior in the case of helical HF membrane designs rather than straight ones and helps to reach the optimum configuration of this innovative design. In addition, this study includes an investigation of the effect of a multi-stage configuration on the desalination system’s thermal performance.

## 2. HF-WGMD System Description

**Figure 1:**
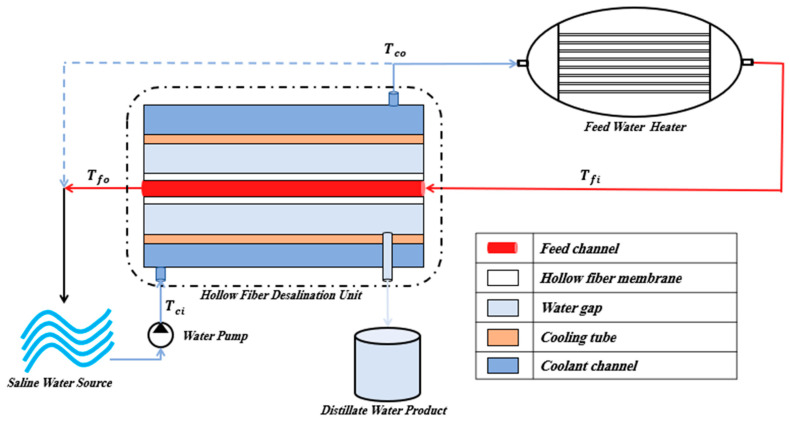
Schematic diagram of HF-WGMD unit with the flow streams.

A typical HF-WGMD desalination unit comprises five essential components, the feed water channel, HF membrane, stagnant water gap, cooling tube, and coolant channel, as shown in Figure 1. The cooling channel serves as an inlet for saline water. This water is pumped by a circulating pump, functioning as a coolant that is responsible for recuperating thermal energy from the water gap. Then, the assigned amount of saline water to be desalinated is heated using an external heating source (feed water heater) while the excess saline water from the cooling channel (denoted by dashed line in Figure 1) is rejected directly to the saline water tank. The hot feed water flows through the feed channel to be distillated using the HF membrane. Finally, the brine is rejected again to the saline water tank, and the distillate water product is collected using the water gap that separates the HF membrane and the cooling tube.

**Figure 2:**
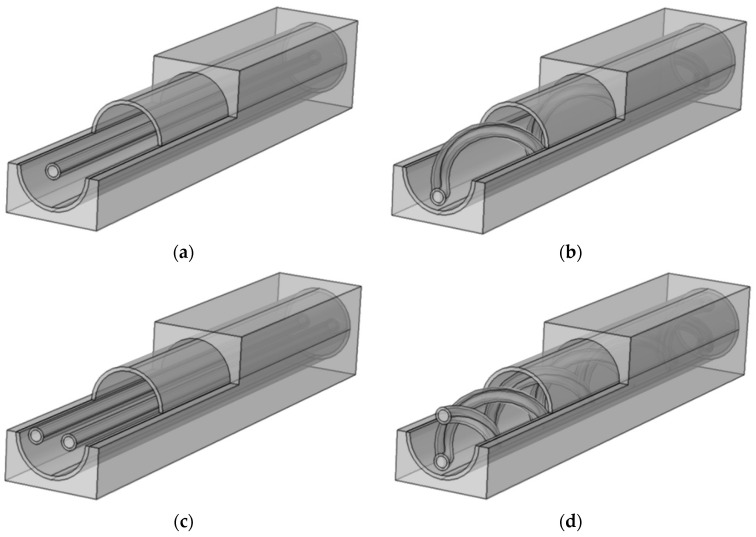
The simulated 3D HF-WGMD units with (**a**) single straight fiber, (**b**) single helical fiber, (**c**) double straight fibers, and (**d**) double helical fibers, all inserted inside the unit cooling tube.

The current study will focus on four distinct HF-WGMD configurations depending on the number and shape of hollow fibers inserted inside the module cooling tube. They include single straight and single helical hollow fibers (depicted in Figure 2a,b, respectively) and double straight and double helical hollow fibers (depicted in Figure 2c,d, respectively).

## 3. Mathematical Model

**Figure 3:**
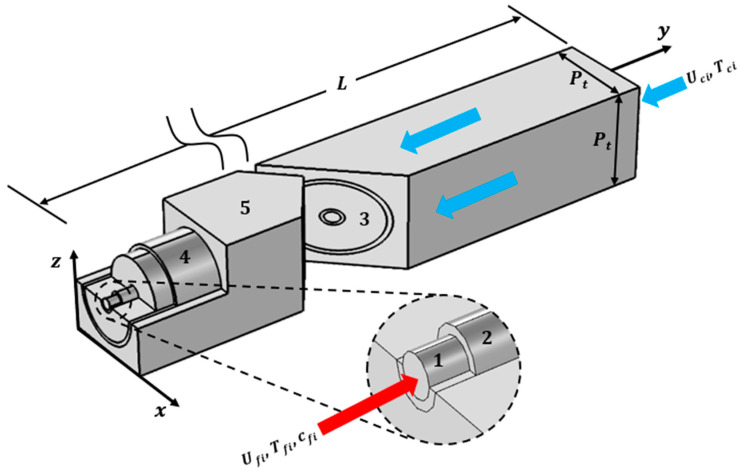
A representation diagram of the 3D simulated HF-WGMD unit. (1) Feed channel, (2) HF membrane, (3) water gap, (4) cooling tube, and (5) coolant channel.

Modeling WGMD involving hollow fiber membranes is a complex task that requires the consideration of the heat and mass transfer, fluid dynamics, and membrane characteristics. Since the helicity of the fibers makes the flow problem three-dimensional, and to reduce the computational effort and time, only a single cooling tube will be considered. COMSOL Multiphysics is a powerful software tool used to simulate and model various physical phenomena, including membrane distillation processes, using hollow fiber membranes. The main five domains that will be simulated in COMSOL are the feed channel, HF membrane, stagnant water gap, cooling tube, and coolant channel. Figure 3 illustrates these five domains. The details of the methodology of the solution will be given in the subsequent sections.

A 3D mathematical model is developed to solve the basic conservation equations of mass, momentum, and energy including the next considerations:

- The feed and coolant flows are laminar and in a counter flow pattern.
- The flow is stagnant in the water gap.
- The HF membrane porosity is uniformly distributed.
- There is no pore wetting of the membrane.
- The MD module’s exterior heat losses are disregarded.
- No fouling occurs on the feed–membrane interface.

The four outer sides of the unit $\left(at\;x=0,\;x=P_{t},\;z=0,\;and\;z=P_{t}\right)$ are considered as symmetry boundary conditions to simulate the periodic inline arrangement pattern (with spacing of $P_{t}$) of the cooling tubes inside the HF-WGMD module shell. Table 1 contains a list of the primary geometrical characteristics and operational circumstances for the simulated unit.

### 3.1. Governing Equations

In this study, the transport phenomena of the mass, momentum, and energy are considered in the five domains of the desalination unit. The water transport in the feed channel via convection and diffusion is considered. The water vapor transport through the HF membrane thickness is modeled using the typical molecular and Knudsen diffusion mechanisms. In addition, the water vapor concentrations are calculated on both the hot and cold interfaces of the membrane depending on the saturation pressures, considering the effects of the local feed water temperature and salinity on the feed–membrane (hot) interface and the local distillate water temperature on the membrane–water gap (cold) interface using the Antoine equation. On the other hand, the heat transport via both conduction and convection is modeled in the feed and coolant channel domains, while conduction only is considered in the HF membrane, stagnant water gap, and cooling tube domains.

#### 3.1.1. Mass Transport Phenomena

The water mass transfer physics is solved for both the feed and HF membrane domains to calculate the local water concentrations. In the feed channel, the local water concentration is calculated by solving the concentration conservation equation, including both the convection and diffusion mechanisms through the feed saline water, as follows:

(1) $$\nabla \cdot \left(D_{w}\nabla c_{f}\right)=\overset{⃑}{u_{f}}\cdot \nabla c_{f}$$

where $D_{w}$ is the water–salt mutual diffusion coefficient, $c_{f}$ is the local water concentration, and $\overset{⃑}{u_{f}}$ is the feed velocity field vector.

On the other hand, only the diffusion mechanism is considered when solving the concentration conservation equation in the HF membrane layer. So, the water vapor molecule diffusion is modeled using Fick’s second law as follows:

(2) $$\nabla \cdot \left(D_{m}\nabla c_{m}\right)=0$$

where $D_{m}$ is the diffusion coefficient of water vapor molecules through the HF membrane pores and $c_{m}$ is the local vapor concentration in the membrane domain.

#### 3.1.2. Momentum Transport Phenomena

The local velocity fields in both the feed and coolant channels are calculated by solving the Navier–Stokes equations. For steady, incompressible, and laminar flows, they can be presented in the vector form as follows [29]:

(3) $$\nabla \cdot \overset{⃑}{u}=0$$

(4) $$\rho \left(\overset{⃑}{u}\cdot \nabla \right)\overset{⃑}{u}=-\nabla p+\mu \nabla^{2}\overset{⃑}{u}$$

where $\overset{⃑}{u}$ is the velocity field vector, ρ is the fluid density, p is the flow local pressure, and μ is the fluid dynamic viscosity for both the feed and coolant flows.

#### 3.1.3. Thermal Energy Transport Phenomena

The local temperature distributions in the entire module domains are obtained by solving the energy conservation equation (feed, HF membrane, water gap, cooling tube, and coolant). The thermal energy transfer via both convection and conduction are considered in the feed and coolant channels’ domains, as follows:

(5) $$\rho C_{p}\left(\overset{⃑}{u}\cdot \nabla \right)T=\nabla \cdot (k\nabla T)$$

where $\overset{⃑}{u}$ is the velocity field vector, ρ is the fluid density, $C_{p}$ is the specific heat, k is the thermal conductivity, and T is the local temperature for both the feed and coolant flows.

Only the heat transfer via conduction is considered in the energy conservation equation solved for the HF membrane, stagnant water gap, and cooling tube metal domains, as follows:

(6) $$\nabla \cdot \left(k\nabla T\right)=0$$

where k is the thermal conductivity and T is the local temperature for the HF membrane, stagnant water gap, or cooling tube metal domains.

Additional equations for the feed and membrane diffusion coefficients and thermal conductivity are used in the mathematical model and coupled with the basic conservation equations, and they are listed in Appendix A.

### 3.2. Boundary Conditions

The external boundaries of the five domains of the simulated desalination unit considered in the current study are listed in Table 2.

Additional boundary conditions used to model the heat source and sink in addition to the vapor concentration on both HF membrane interfaces are listed in Appendix A.

### 3.3. Solution Procedure

By utilizing the finite element method made available by the COMSOL Multiphysics software, the mathematical model given in the current work is solved. The transport phenomena in the five domains of the various desalination units considered in Figure 2 are represented using the heat, mass, and laminar flow physics packages inside COMSOL. Furthermore, user-defined equations such as the feed and HF membrane diffusion coefficients, the modified Antoine equations (see Appendix A), and the thermophysical and temperature-dependent properties are added and coupled with the main physics equations. For the five domains of the desalination unit, the system equations are fully coupled and simultaneously solved while using the boundary conditions and the assumptions indicated above.

### 3.4. Performance Evaluation Parameters

In the current study, four specific parameters are used to assess and compare the performance of the simulated desalination units. The CFD model is adopted to calculate the desalination unit’s specific productivity (SP), specific thermal energy consumption (STEC), percentage of thermal energy recovery (%TER), and gain output ratio (GOR). The specific productivity is defined as the daily water productivity produced per cubic meter of the desalination unit volume in m_w_^3^/(m_du_^3^ day) and can be determined as follows:

(7) $$SP=\frac{24J_{w}A_{m}}{{{1000P}_{t}}^{2}L}$$

where $J_{w}$ is the water product flux in L/(m^2^ h), $A_{m}$ is the total HF membrane’s effective area inside the cooling tube in m^2^, and $P_{t}$ and L are the cooling tube spacing and module’s effective length in m, respectively, as illustrated in Figure 3.

The specific thermal energy consumption is defined as the thermal energy consumed by the desalination unit per unit mass of the produced water in kWh/kg_w_ and can be determined as follows:

(8) $$STEC=\frac{\dot{m_{f}}C_{p_{f}}(T_{fi}-T_{co})}{J_{w}A_{m}}$$

where $\dot{m_{f}}$ is the feed mass flow rate in kg/s, $C_{p_{f}}$ is the feed fluid’s specific heat in kJ/(kg°C), $T_{fi}$ is the feed inlet temperature in °C, and $T_{co}$ is the coolant outlet temperature in °C.

Furthermore, WGMD provides an advantage of recovering the heat that is lost from the feed flow due to evaporation and conduction by using the saline water as the module coolant, as illustrated in Figure 1. So, the model is adopted to calculate the percentage of recovered energy of the total required heating energy as follows:

(9) $$\%TER=\frac{T_{co}-T_{ci}}{T_{fi}-T_{ci}}$$

where $T_{ci}$ is the coolant inlet temperature.

Finally, the GOR is used to indicate the thermal energy efficiency of the WGMD system, which can be defined as the ratio of thermal energy consumed due to evaporation to the heating energy consumed in the feed water heater. It can be calculated as follows:

(10) $$GOR=\frac{J_{w}A_{m}h_{fg}}{\dot{m_{f}}C_{p_{f}}(T_{fi}-T_{co})}$$

where $h_{fg}$ is the water latent heat of vaporization.

### 3.5. Grid Independence Test

The selection of physics-controlled mesh in COMSOL Multiphysics is used to generate three different grid levels. The grids are tested for the five domains of a desalination unit with a single straight fiber to minimize the discretization error produced due to the numerical solution and, at the same time, to reduce the computational efforts. As indicated in Table 3, using a number of elements above 2,411,325 elements has no significant effect on the water output flux or feed outlet temperature, with maximum change of 0.55% when using a higher number of elements. So, using Grid 2 is satisfactory for the current simulation process, resulting in an element average quality above 0.8.

## 4. Results and Discussion

### 4.1. Experimental Validation of CFD Model

**Figure 4:**
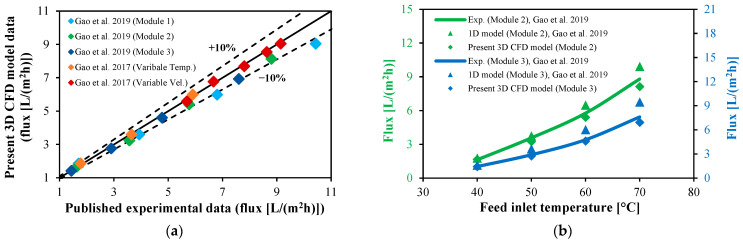
Experimental validation. (**a**) Numerical flux calculated using the 3D CFD model against flux produced by different experimental modules [14,28] at the same operating conditions. (**b**) Output flux comparison between the current 3D CFD model and the 1D model proposed by [28] for the same modules with multi-fibers inside the cooling tube.

The CFD model is validated using the experimental data obtained by Gao et al. [14,28]. It is worth mentioning that the entire experiments were performed on a straight HF-WGMD module that consists of eight High-Density Polyethylene (HDPE) cooling tubes inside a PE pipe used as the module shell. The different operating conditions and module lengths of the experimental WG modules are listed in Table 4. The numerical and experimental fluxes are compared for each module, and most test cases have an absolute deviation of less than 10%, as illustrated in Figure 4a, whereas the calculated average percent of deviation for all test cases is around −3%.

On the other hand, the superiority of the current 3D CFD model is evaluated with that of the 1D model introduced by Gao et al. [28] in the case of multi-fibers inserted in the module’s cooling tubes, and both are compared with the experimental results of modules 2 and 3 of Gao et al. [14,28]. As presented in Figure 4b, for module 2 and a feed inlet temperature of 70 °C, the 1D model has a maximum deviation in the module flux of 12.8%, whereas only a −7.6% deviation is observed in the case of using the current 3D model. In the case of modeling module 3, the 1D model overestimates the module output flux by 26.3% (case of maximum percentage of deviation), whereas the 3D model underestimates the module flux by only 3.5%, with both at a 60 °C feed inlet temperature.

### 4.2. CFD Simulation of Helical HF-WGMD

In the current work, a single cooling tube of the whole HF-WGMD module is considered with single and double hollow fibers inserted in the shape of straight normal fibers or helical ones. In the next sections, CFD figures are extracted to provide flow visualization data for the velocity, temperature, and concentration contours for all domains of the simulated desalination units. The CFD figures are used to help clarify the effects of different operating conditions, such as the feed and coolant stream inlet velocities and the feed inlet temperatures, on the desalination units’ performances. In addition, the geometrical parameters of hollow fibers, such as the number of helical turns in single and double fibers inside the cooling tube, are included in the CFD study.

#### 4.2.1. Effect of Feed Inlet Velocity on Feed Salinity and Temperature

The feed stream velocity is one of the major factors that influence both the concentration and temperature polarization at the feed–membrane interface in the MD module. As it is obvious in the modified Antoine equation, both the salt concentration and feed water temperature at the interface directly control the saturation pressure, and hence, the membrane’s hot side concentration, which controls the water vapor diffusion process through the membrane pores.

**Figure 5:**
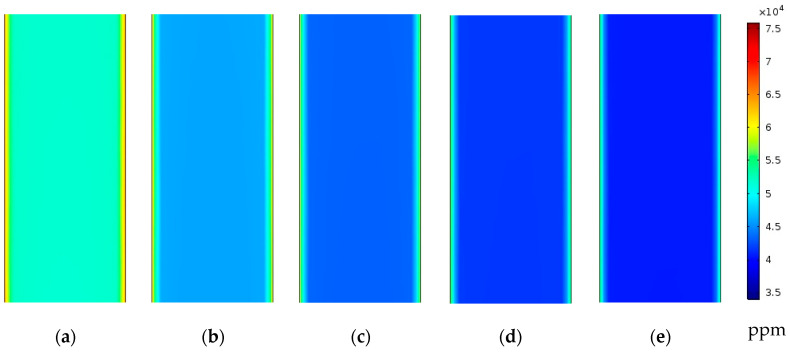
Feed water salinity contours at middle of xy half section in the feed channel of desalination unit with single straight fiber at $U_{ci}=0.21\;m/s\;and\;T_{fi}=70\;{}^{\circ}C$; (**a**) $U_{fi}=0.29\;m/s$, (**b**) $U_{fi}=0.58\;m/s$, (**c**) $U_{fi}=0.87\;m/s$, (**d**) $U_{fi}=1.16\;m/s$, and (**e**) $U_{fi}=1.45\;m/s$.

The effect of the feed inlet velocity on the concentration polarization phenomena is considered by studying five laminar flow velocities of 0.29, 0.58, 0.87, 1.16, and 1.45 m/s (461<Re<2300), while the coolant inlet velocity is kept at 0.21 m/s and the feed inlet temperature is kept at 70 °C for the desalination unit with the single straight fiber inside the cooling tube. As illustrated in Figure 5, considering higher velocities at the feed channel inlet reduces the salt concentrations at the feed–membrane interface, which enhances the water vapor concentration, and hence, the driving force for vapor diffusion. The average salt concentration at the feed–membrane interface is reduced from 65,401 ppm to 61,552 ppm when increasing the feed inlet velocity from 0.29 m/s to 0.87 m/s, and a much higher reduction is achieved when increasing the inlet velocity to 1.45 m/s, at which the average salt concentration is at a minimum value of 58,945 ppm; all concentrations are measured at a fiber middle length, as depicted in Table 5.

**Figure 6:**
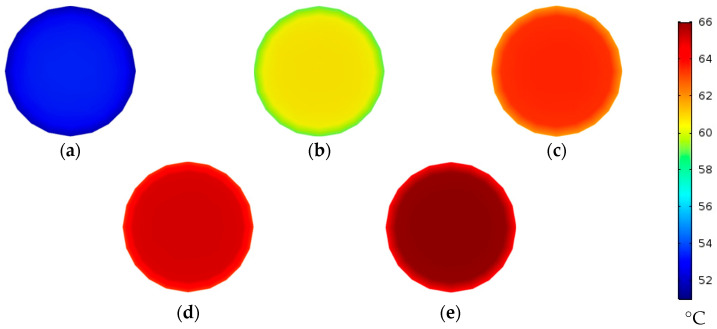
Feed water temperature contours at zx cross section at middle of fiber length (y=250 mm) of desalination unit with single straight fiber at $U_{ci}=0.21\;m/s\;and\;T_{fi}=70\;{}^{\circ}C$; (**a**) $U_{fi}=0.29\;m/s$, (**b**) $U_{fi}=0.58\;m/s$, (**c**) $U_{fi}=0.87\;m/s$, (**d**) $U_{fi}=1.16\;m/s$, and (**e**) $U_{fi}=1.45\;m/s$.

On the other hand, the effect of the feed inlet velocity on the temperature polarization is studied at the five feed velocities, as shown in Figure 6. It should be evident that the increased feed flow velocity results in higher bulk feed temperatures, which raises the feed–membrane interface temperatures. The average temperature at the feed–membrane interface measured at the middle of the fiber length is increased by around 10 °C when tripling the feed inlet velocity from 0.29 m/s to 0.87 m/s, while the interface temperature reaches a maximum value of 64.2 °C at a 1.45 m/s feed velocity, as depicted in Table 5.

#### 4.2.2. Effect of Coolant Inlet Velocity on Water Gap Temperature

The cooling channel functions as the module heat sink in the WGMD systems, providing enough cooling for the stagnant distillate water gap through the cooling tube. This is carried out to maintain a lower temperature and lower saturation pressure at the membrane–WG interface to improve the vapor diffusion through the membrane pores. As a result, the cooling scheme might be greatly impacted by the coolant stream’s velocity.

**Figure 7:**
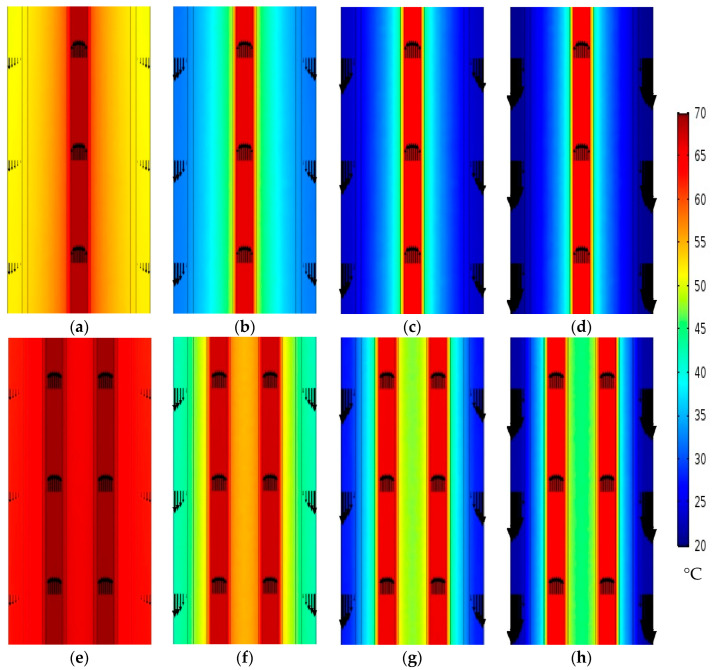
Temperature contours at middle of xy section in desalination unit at $U_{fi}=1.16\;m/s\;and\;T_{fi}=70\;{}^{\circ}C$ with single fiber [(**a**) $U_{ci}=0.0031\;m/s$, (**b**) $U_{ci}=0.0125\;m/s$, (**c**) $U_{ci}=0.05\;m/s$ and (**d**) $U_{ci}=0.21\;m/s$], and double straight fibers [(**e**) $U_{ci}=0.0031\;m/s$, (**f**) $U_{ci}=0.0125\;m/s$, (**g**) $U_{ci}=0.05\;m/s$ and (**h**) $U_{ci}=0.21\;m/s$]. The arrows represent the axial velocity magnitudes relative to each other.

Different coolant inlet velocities of 0.0031, 0.0125, 0.05, and 0.21 m/s are tested in this section for modules with both single and double straight fibers, as illustrated in Figure 7. In both the single- and double-fiber situations inside the module cooling tube, it is evident that providing higher coolant velocities dramatically lowers the WG temperatures.

The WG average temperature can be reduced from 56.4 °C to 41.6, 35.2, and 33.2 °C when elevating the coolant stream’s inlet velocity from 0.0031 m/s to 0.0125, 0.05, and 0.21 m/s, respectively, in the case of using a single fiber inside the module’s cooling tube. However, the WG temperature reduces from 64.7 °C to 50.6, 41.4, and 38.1 °C in the case of double fibers, and at the same coolant inlet conditions, all data are measured at the middle cross section of the desalination unit length, as depicted in Table 6.

On the other hand, it can be noticed that at the same coolant inlet velocity, the WG temperatures achieve higher values in the case of using double fibers rather than when using a single fiber due to the higher distillate productivity. As shown in Table 6, when using a single fiber, the WG average temperatures are 36.1, 35.2, and 33.5 °C at the cross sections located 1125, 250, and 375 mm from the inlet, while higher values of 44.6, 41.4, and 39.7 °C are measured at the same locations when using double fibers, considering a coolant inlet velocity of 0.05 m/s for both cases. By using more packed fibers inside the module’s cooling tube, it becomes more necessary to improve the cooling scheme offered by the cooling channel by pumping more coolant into the cooling channel.

#### 4.2.3. Effect of Using Helical Hollow Fibers on the Water Gap Temperature

The proposed extending hollow fiber lengths using helical configurations are examined for both single and double fibers when 10, 20, 30, 40, and 50 turns are used, and they are compared with the straight fiber scheme.

**Figure 8:**
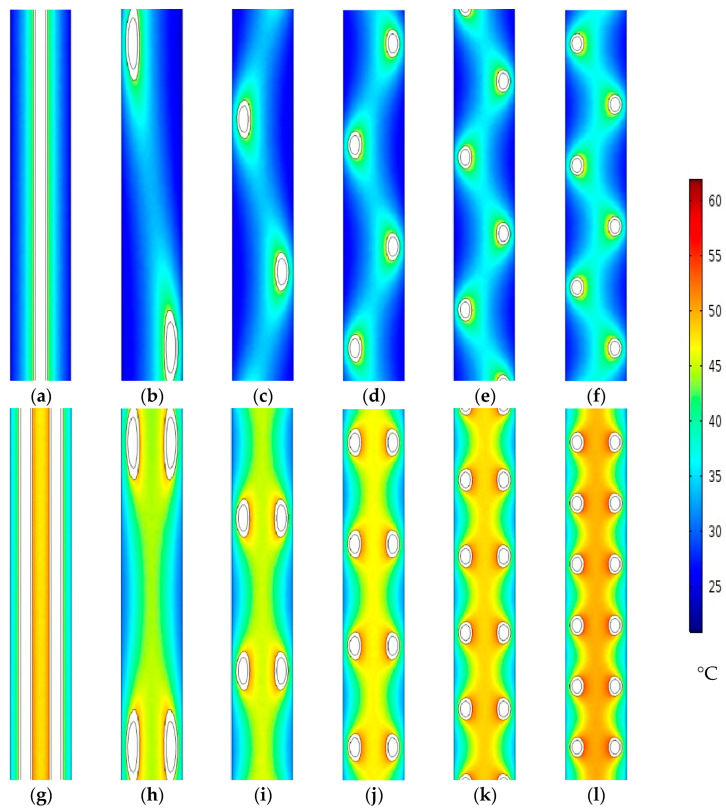
Water gap temperature contours at middle of xy section at $U_{fi}=1.16\;m/s,\;U_{ci}=0.05\;m/s,\;and\;T_{fi}=70\;{}^{\circ}C$ for desalination unit with single (**a**) straight fiber with (**b**) 10, (**c**) 20, (**d**) 30, (**e**) 40, and (**f**) 50 turns of helical fibers and double (**g**) straight fibers with (**h**) 10, (**i**) 20, (**j**) 30, (**k**) 40, and (**l**) 50 turns of helical fibers.

In general, using helical fibers with a greater number of turns applies more heating load to the water gap of the MD module, as illustrated in Figure 8. Increasing the helical HF turns increased the distillated water productivity, which generated more condensation heat in the water gap channel. This is also combined with the increased heat loss caused by the heat conduction from the longer HF membrane (higher surface area) to the water gap channel. As a result, the anticipated enhancement in the flux acquired by the helical form in the feed channel was gradually eliminated by the temperature rise in the water gap when using a higher number of HF turns. Therefore, the module output flux can be negatively affected due to higher saturation pressures applied to the membrane–WG interface, especially in the case of double helical fibers. Figure 8 shows the accumulated hot layers of water gap near the membrane interface in both single and double fibers, especially near the core of the module water gap.

The heat transfer in the water gap depends mainly on the conduction heat mechanism (as the water is almost stagnant). Therefore, the shorter the distance to the coolant tube, the less heat transfer resistance there will be. This points toward a positive effect on heat transfer from the helical HF form as it is closer to the coolant tube. This was more obvious when a smaller number of 10 helical turns was examined, as can be seen in Figure 8b, which resulted in a lower WG temperature. On the other hand, the heat transfer at the interface between the HF membrane and water gap increases by increasing the HF length due to the heat generated by permeate condensation and the heat transferred via conduction. This was more obvious in Figure 8 at higher HF turns, especially with the double HF compared to the single ones.

As presented in Table 7, the WG bulk mean temperature is slightly decreased from 31.4 to 30 °C when using 10 turns of a single helical fiber instead of a straight normal fiber. The drop in the WG temperature can be due to the narrower distance between the fiber and cooling tube in a helical fiber configuration, as shown in Figure 8b. However, the WG temperature increases again up to 30.5, 31.2, 32, and 32.9 °C for a single helical fiber with 20, 30, 40, and 50 turns, respectively, due to the increased heating load provided by the extended fiber length, as depicted in Table 7.

On the other hand, the WG bulk temperature increases from 38.2 °C for double straight fibers to 38.4, 39.2, 40.4, 41.5, and 42.9 °C for 10, 20, 30, 40, and 50 turns of double helical fibers, respectively. This increase in the WG temperature can be justified as the double fibers already occupy most of the water gap, so no significant effect can be realized by the cooling tube.

By examining the difference in the water vapor concentration across the membrane layer, it is possible to assess the overall impact of the proposed helical fiber configuration on the diffusion force. The average values for the difference in water vapor concentration between the feed and WG membrane interfaces are shown in Table 8. When utilizing a helical fiber with 20 turns, maximums of 4.51 mol/m^3^ and 4.116 mol/m^3^ are attained in the case of single and double fibers, respectively. Due to the significant accumulated heating load caused by the double fiber configuration, the peak concentration difference in the case of a single fiber is bigger than that of double fibers.

#### 4.2.4. Effect of Feed Water Inlet Temperature on the Vapor Concentration on Both Sides of the HF Membrane

**Figure 9:**
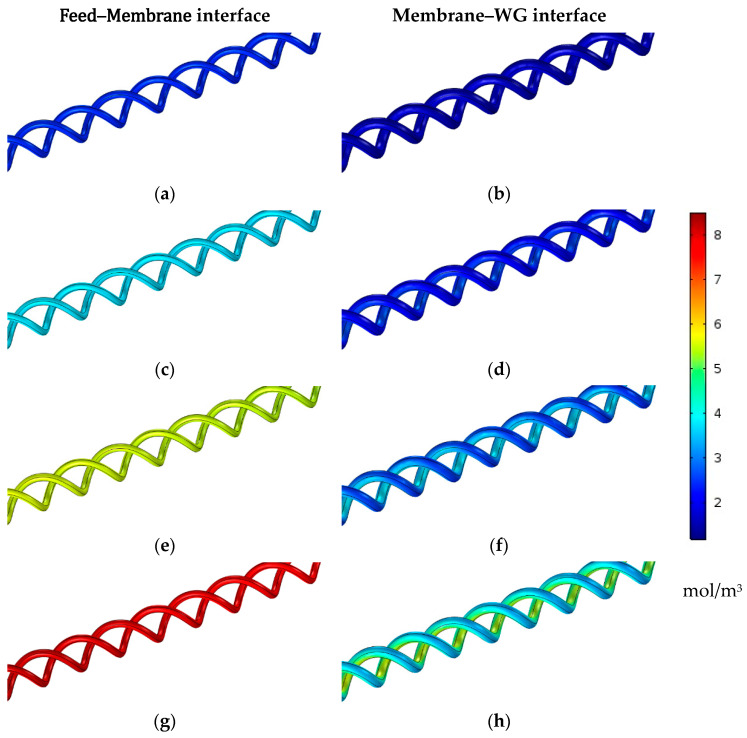
Water vapor concentration contours on both HF membrane interfaces with 50 turns of double helical fibers at $U_{fi}=1.16\;\;m/s\;and\;{\;U}_{ci}=0.05\;m/s$. (**a**,**b**) $T_{fi}=40\;{}^{\circ}C$, (**c**,**d**) $T_{fi}=50\;{}^{\circ}C$, (**e**,**f**) $T_{fi}=60\;{}^{\circ}C$, and (**g**,**h**) $T_{fi}=70\;{}^{\circ}C$.

The effect of the feed water inlet temperature on the water vapor concentration on both membrane interfaces is investigated at different temperature levels. Figure 9 illustrates the vapor concentration contours distributed at the feed–membrane and membrane–WG interfaces for a module of double helical fibers with 50 turns at different feed inlet temperatures. The concentration on both membrane interfaces increases as the temperature rises, as does the water vapor saturation pressure.

On the other hand, Table 9 lists the average values of water vapor concentrations on both membrane interfaces in addition to their differences to illustrate the net effect of the feed inlet temperature level on the vapor diffusion force. The water vapor concentration at the feed–membrane interface is around 2.445 mol/m^3^ at a 40 °C feed temperature and enhances by 54, 129.5, and 232.2% at 50, 60, and 70 °C feed inlet temperatures, respectively.

The water vapor concentration increases at the membrane–WG interface, but at a lower rate with an increasing feed inlet temperature. It increases by only 35.6, 87.1, and 162.4% when elevating the feed temperature from 40 °C to 50, 60, and 70 °C, respectively. Therefore, the feed inlet temperature level has a positive and significant effect on the water vapor penetration across the HF membrane layer. The average concentration difference across the membrane increases from 0.763 mol/m^3^ to 1.485, 2.465, and 3.709 mol/m^3^ when increasing the feed inlet temperature from 40 °C to 50, 60, and 70 °C, respectively, as depicted in Table 9.

### 4.3. Parametric Studies

The impacts of the operational and geometrical parameters on the desalination unit’s performance with the HF membrane’s helical designs is examined in the following sections. To choose module flow velocities that maximize the flux and productivity with the least amount of hydraulic loss, parametric studies on the feed and coolant input velocities are conducted.

#### 4.3.1. Effect of Feed Flow Velocity on the Water Output Flux

**Figure 10:**
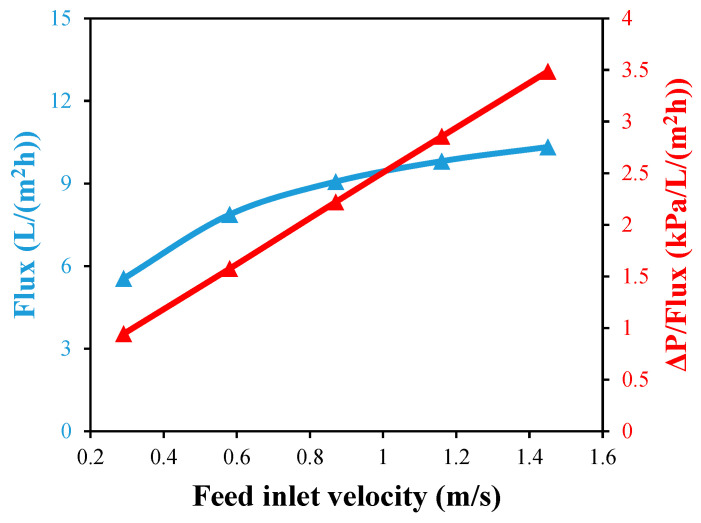
Water output flux and pressure drop per unit flux against feed inlet velocity with single straight fiber module at $T_{fi}=70\;{}^{\circ}C$ and $U_{ci}=0.21\;m/s$.

For a desalination unit with a single straight fiber, as illustrated in Figure 10, the effect of five feed inlet velocities in the laminar flow region on the module output flux is examined using a feed inlet temperature of 70 °C and a coolant inlet velocity of 0.21 m/s. An increasing feed velocity has a positive impact on the output flux due to the reductions in the temperature and concentration polarizations. When the feed velocity is doubled from 0.29 to 0.58 m/s, the flux is increased by about 41.8%, but it is only increased by 24.7% when it is doubled again to 1.16 m/s.

Figure 10 shows the effect of the feed inlet velocity on the HF pressure drop per unit of flux. As the feed velocity in the feed channel rises, the pressure drop also increases. When the feed velocity is increased from 0.29 to 0.58 m/s, the pressure drop per unit flux increases by about 68%, and an additional 81% is added when the velocity is increased again to 1.16 m/s. Hence, it is necessary to compromise the feed velocity to magnify the water output flux with reasonable hydraulic pressure losses. In this case, a feed velocity of 1.16 m/s is selected to be the most reasonable value in the current study. Since the water output flux at a 1.16 m/s feed velocity is 9.81 L/(m^2^ h), the authors choose to forego an additional 5.3% of flux at 1.45 m/s to prevent an additional 22% of pressure loss per unit of produced flux.

#### 4.3.2. Effect of Coolant Flow Velocity and Number of Helical Turns on the Water Output Flux

**Figure 11:**
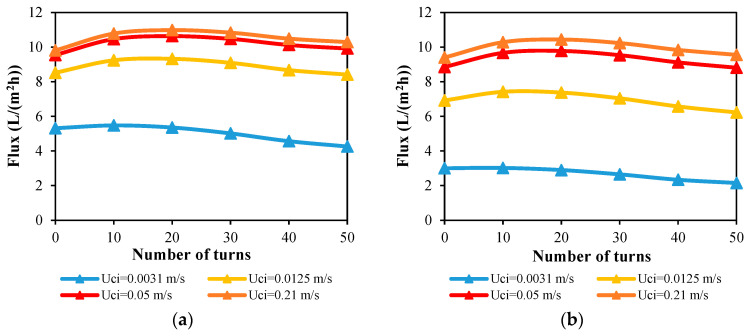
Water output flux against number of HF helical turns at different coolant inlet velocities and $U_{fi}=1.16\;m/s\;and\;T_{fi}=70\;{}^{\circ}C$ for desalination units with (**a**) single helical fiber and (**b**) double helical fibers.

For desalination units with single and double fibers in straight and helical forms, the impact of the module coolant velocity on the output flux is investigated and reported at a 70 °C feed inlet temperature and a 1.16 m/s feed inlet velocity. By comparing Figure 11a,b, the flux produced by the single helical HF membrane module is higher than the double helical one at any helical turns of the same coolant velocity. For both configurations, the maximum fluxes can be achieved at a number of turns between 10 and 20.

The flux rises from 9.81 L/(m^2^ h) for a single straight fiber to a peak value of 10.98 L/(m^2^ h) for a single helical fiber with 20 turns at a velocity of 0.05 m/s in the coolant inlet, which can be explained by the helical fiber approaching from the module’s cooling tube in a helical configuration, as discussed in Section 4.2.3. At 50 helical turns, the flux then decreases once again to 9.92 L/(m^2^ h) as a result of the greater heating loads imposed on the WG by the longer fiber length. The same pattern is observed for double fibers, where the water flux is increased by 10.4% at 20 helical turns at a 0.05 m/s coolant velocity and lowered once again at higher numbers of helical turns (Figure 11b). At the minimum velocity of 0.00031 m/s, however, the straight HF fiber module produces the highest flux.

**Figure 12:**
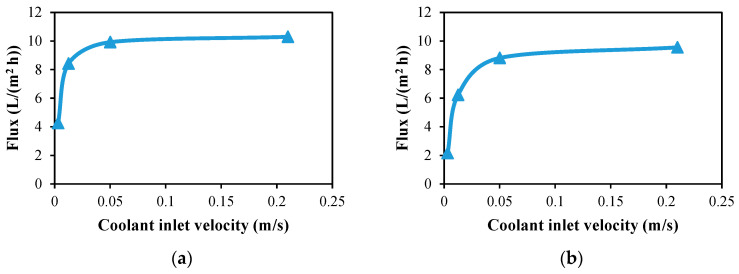
Water output flux against coolant inlet velocity at $U_{fi}=1.16\;m/s\;and\;T_{fi}=70\;{}^{\circ}C$ for a desalination unit with 50 turns of helical fiber; (**a**) single and (**b**) double.

The water flux produced by 50 turns of single and double helical fiber modules is plotted against the coolant inlet velocity in Figure 12. At a 0.05 m/s coolant velocity, the water flux increases to 9.92 L/(m^2^ h) for single fibers and 8.82 L/(m^2^ h) for double fibers, but at a 0.21 m/s coolant inlet velocity, only 4% to 8% of the flux can be added. So in the performance evaluation of the desalination units in the next sections, 0.05 m/s will be set as the coolant inlet velocity.

#### 4.3.3. Effect of Feed Temperature on the Flux and Productivity

**Figure 13:**
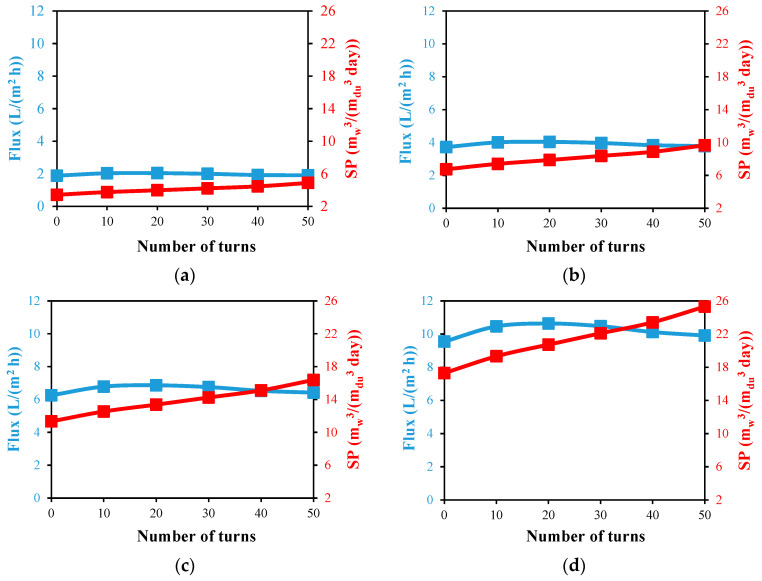
Water output flux and specific productivity against number of HF helical turns for desalination unit with single fiber at $U_{fi}=1.16\;m/s\;and\;\;U_{ci}=0.05\;m/s$; (**a**) $T_{fi}=40\;{}^{\circ}C$, (**b**) $T_{fi}=50\;{}^{\circ}C$, (**c**) $T_{fi}=60\;{}^{\circ}C$, and (**d**) $T_{fi}=70\;{}^{\circ}C$.

In Figure 13 at various helical turn numbers, the impacts of the feed inlet temperatures of 40, 50, 60, and 70 °C on the flux and specific productivity of a desalination module of a single helical HF membrane is shown. The inlet velocities for the feed and coolant are set at 1.16 and 0.05 m/s, respectively.

The best distilled water flux is always at 20 helical turns and increases significantly from a maximum of 2.04 L/(m^2^ h) at a feed temperature of 40 °C to maximums of 4.04, 6.87, and 10.64 L/(m^2^ h) at feed temperatures of 50, 60, and 70 °C, respectively. At higher feed temperatures, the effect of the number of turns on the produced flux is more obvious. When comparing the flux at 20 turns with the reference module of a straight HF membrane at 70 °C, the highest increase of 11.4% is attained, as noticed in Figure 13d, and only a 7.9% flux increase is achieved at a 40 °C feed temperature, as noticed in Figure 13a. The flow in the turning pass associated with the helical HF design increases the flow mixing and reduces the temperature and salt concentration polarizations at the boundary layer, which, in turn, can enhance the produced flux. However, the accumulated heat in the water gap channel works on eliminating these enhancements in the flux, especially at higher numbers of turns.

The specific productivity of the distillation module improves with the number of helical turns at a particular feed temperature; for feed temperatures of 40 °C and 70 °C, respectively, it increases by 42.6% and 46.2% when 50 turns are merged to the module compared to the reference straight one. From 50 turns of single helical modules at a 70 °C feed temperature, a maximum specific productivity of 25.3 m_w_^3^/day per cubic meter of desalination unit is produced, as shown in Figure 13d. This demonstrates the significant effect of increasing the fiber length using the proposed helical configuration without affecting the total volume of the module.

**Figure 14:**
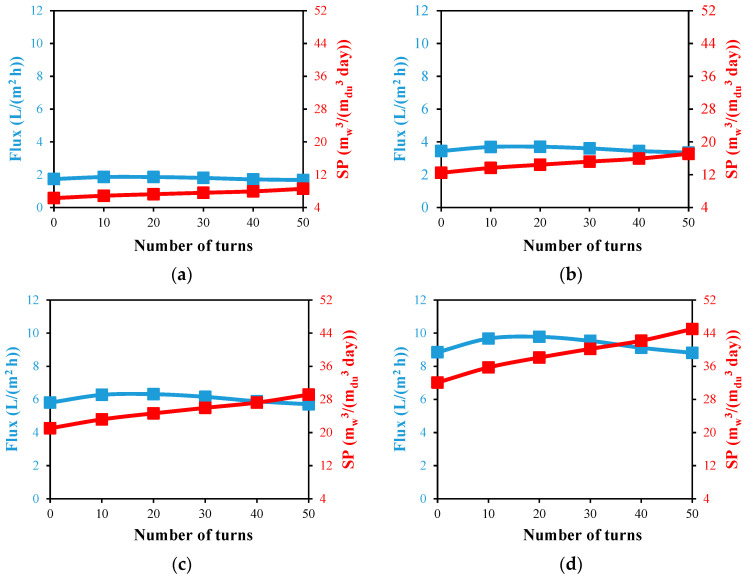
Water output flux and specific productivity against number of HF helical turns for desalination unit with double fibers at $U_{fi}=1.16\;m/s\;and\;U_{ci}=0.05\;m/s$; (**a**) $T_{fi}=40\;{}^{\circ}C$, (**b**) $T_{fi}=50\;{}^{\circ}C$, (**c**) $T_{fi}=60\;{}^{\circ}C$, and (**d**) $T_{fi}=70\;{}^{\circ}C$.

Figure 14 shows the impact of utilizing a double helical HF membrane configuration on the module flux and productivity at various feed temperatures and turning counts. The double straight HF membrane arrangement is represented in this figure by the zero number of turns. Due to the higher heat that has accumulated in the water gap, the double helical HF membrane’s maximum flux is attained at around 20 turns, with values that are marginally less than those obtained from the single helical ones. At a 70 °C feed temperature and 20 turns, the maximum flux of 9.78 L/(m^2^ h) is reached, which is smaller than the corresponding one with a single helical case of 8.07%.

At a 70 °C feed temperature and 50 turns, the double helical configuration produces the highest specific productivity of 45 m_w_^3^/(m_du_^3^ day), which is 40.3% higher than the specific productivity of the double straight configuration at the same feed temperature. When compared to the single helical design under the identical conditions, as shown in Figure 13d and Figure 14d, this maximum productivity is significantly greater by 78%. Due to the noticeable reduction in fluxes produced by double configurations brought on by the increased heating load applied to the water gap, as mentioned in Section 4.2.3, the desalination unit’s specific production of a double helical design is observed not to be doubled over the modules of a single helical design. However, the double helical design will be preferable when the desalination unit’s compactness is the primary consideration.

#### 4.3.4. The Effect of Feed Temperature on the Thermal Performance

In general, increasing the number of helical HF turns increased the HF length, which reduced the STEC in two ways: (i) by utilizing more thermal energy from the same feed temperature, expressed by a lower outlet feed temperature, and (ii) by recovering more thermal energy via the coolant, which directly reduced the heat added by the feed water heater. Finally, the productivity increased by increasing the number of turns combined with less energy consumption in the module, which resulted in a reduction in the STEC.

**Figure 15:**
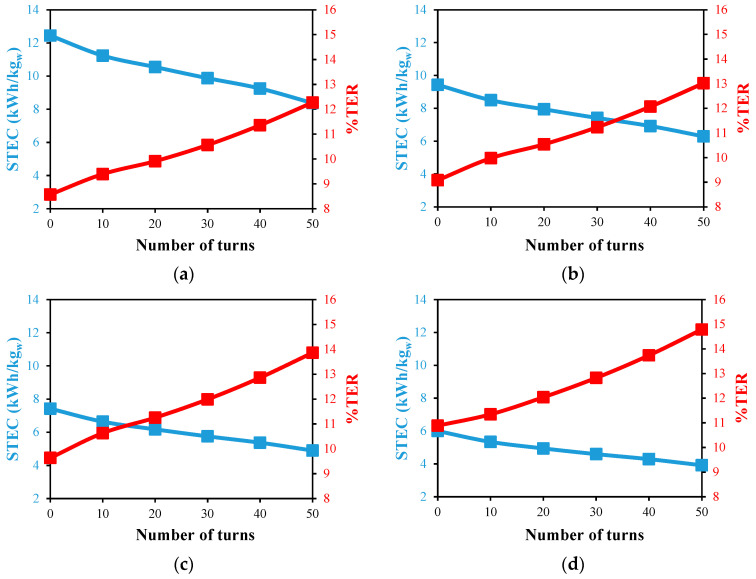
STEC and %TER against number of HF helical turns for desalination unit with single fiber at $U_{fi}=1.16\;m/s\;and\;\;U_{ci}=0.05\;m/s$; (**a**) $T_{fi}=40\;{}^{\circ}C$, (**b**) $T_{fi}=50\;{}^{\circ}C$, (**c**) $T_{fi}=60\;{}^{\circ}C$, and (**d**) $T_{fi}=70\;{}^{\circ}C$.

**Figure 16:**
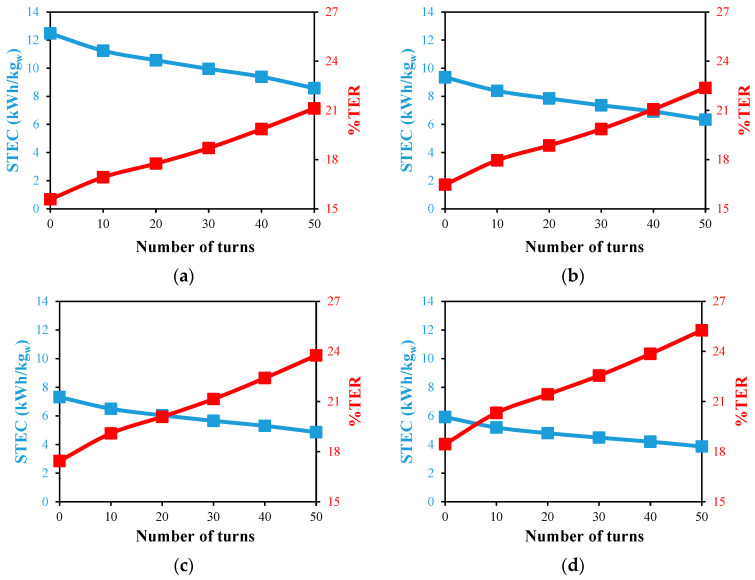
STEC and %TER against number of HF helical turns for desalination unit with double fibers at $U_{fi}=1.16\;m/s\;and\;U_{ci}=0.05\;m/s$; (**a**) $T_{fi}=40\;{}^{\circ}C$, (**b**) $T_{fi}=50\;{}^{\circ}C$, (**c**) $T_{fi}=60\;{}^{\circ}C$, and (**d**) $T_{fi}=70\;{}^{\circ}C$.

Figure 15 and Figure 16 for the single and double designs, respectively, show how the use of helical HF membrane designs affects the energy consumption of the distillation module. In general, as the feed temperature rises, the STEC of both systems drops. That can be explained by the desalination units’ better productivity combined with the lower enthalpy of vaporization of feed water at high feed temperatures. The best STEC of 3.9 kWh/kg_w_ is attained at a 70 °C feed temperature and 50 single helical turns with a positive reduction of 35% in comparison to the reference case, as illustrated in Figure 15d. When the source of the feed is limited to 40 °C [30], the use of 50 single helical turns favorably reduces the STEC by 32.6%, as shown in Figure 15a. Similar findings are obtained for the STEC values in the case of the double helical design, as illustrated in Figure 16. This demonstrates the practical uses for HF in a helical form in the cooling tubes of the WGMD systems to lower the energy consumption and unit size.

In comparison to the reference situation of zero turns, Figure 15 and Figure 16 illustrate the impact of the HF membrane’s helical spiral count on the percentage of thermal energy recovery (%TER). At a particular feed temperature, increasing the number of turns dramatically raises the %TER. According to Figure 15a,b, the largest increase in the %TER of 43.2% is achieved at a feed temperature of 40 °C, while a feed temperature of 70 °C results in an increase of 35.8%. For the double helical design with 50 turns, as shown in Figure 16, significant increases of 35.6% and 36.9% are also attained at 40 and 70 °C feed temperatures. These results showed that the heat recovery within the WGMD modules is supported by the insertion of the HF in a helical twisted form.

#### 4.3.5. The Effect of Multi-Stages Arranged in Series on the System Thermal Performance

To investigate the potential of the multi-stage in enhancing the thermal performance of the WGMD unit, distillation modules are arranged in series, with each module consisting of a single helical HF membrane of 50 turns. In this part, the feed and coolant inlet velocities, as well as the temperature, are set to 1.16 and 0.05 m/s, respectively, and 70 °C. The developed CFD model is extended to simulate up to three stages in series as the workstation capacity limits a further increase in the stages. In this arrangement, saline water is used as a coolant and enters the first stage’s cooling channel before leaving the last stage and starting the heating process. Then, in a counter-flow arrangement with the cooling stream, saline water enters the feed channel of the last stage as a feed and flows to the first stage.

**Figure 17:**
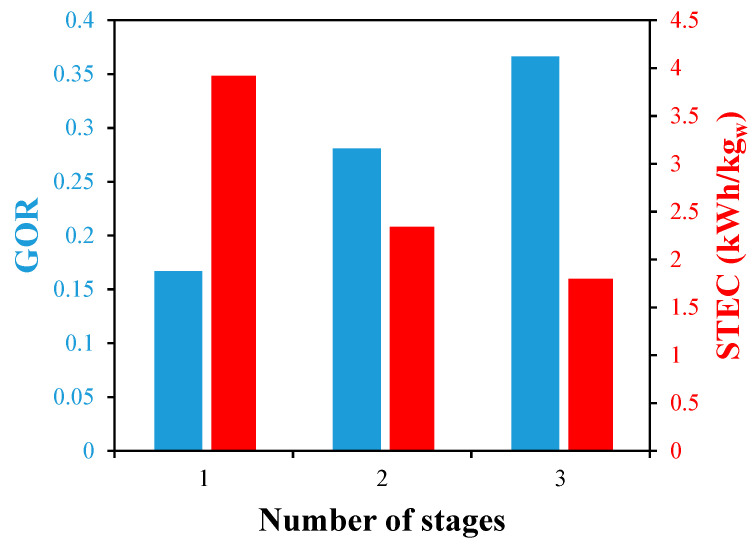
The effect of multi-stages in series on the GOR and STEC of 50 turns of single helical fiber desalination units at $U_{fi}=1.16\;m/s,\;U_{ci}=0.05\;m/s$, and $T_{fi}=70\;{}^{\circ}C$.

According to Figure 17, adding additional stages in series improves the system’s GOR because more heating energy is used to produce more distillate water. The temperature of the rejected brine is lowered by using a longer total HF membrane, which lowers the thermal energy loss. When using three stages in line, the GOR of the desalination unit rises from 0.17 for the single stage to 0.37, which is an increase of 117.6%. The potential of reducing the WGMD’s energy usage with the helical HF membrane design would be higher since more stages are projected to be added. Figure 17 also demonstrates how the unit’s overall STEC decreased favorably from 3.9 kWh/kg_w_ for the single-stage unit to 1.8 kWh/kg_w_ for the three-stage unit, representing a 53.8% decrease in the STEC.

**Figure 18:**
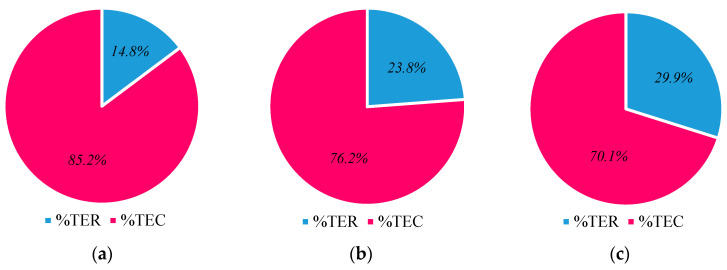
Percentage of thermal energy recovered of (**a**) one, (**b**) two, and (**c**) three single helical fiber desalination units with 50 turns fitted in series at $U_{fi}=1.16\;m/s,\;U_{ci}=0.05\;m/s$, and $T_{fi}=70\;{}^{\circ}C$.

This study also provides information on the percentage of TER in multi-stage systems, as seen in Figure 18. When employing a three-stage unit instead of a single-stage unit, the %TER increased from 14.8% to 29.9%. This is because the %TER rises as the number of stages increases, which will be improved further with more stages.

## 5. Practical Considerations and Future Prospects

The turning of the HF into a helical form was made possible in many previous studies for different applications [31,32,33,34,35], and it was introduced for the first time in this study for the water gap membrane distillation modules. A number of designs and fabrication techniques for helical hollow fiber modules were discussed by Wan et al. [36]. Also, the HF in helical forms can be produced using a spinning technique as demonstrated by Yucel et al. [35]. In this study, the discussion was based on the results of a validated 3D-CFD model, and an explanation that can be used to produce a helical HF by turning them with the help of a perforated tube is given in Appendix A by using techniques discussed in reference [36].

In typical studies that only have straight HF designs, the same length is used, which means increasing the flux directly increases the module productivity, which is the main target. Therefore, the advantage of any new design can be seen by increasing the flux. In this study, however, a fixed module dimension was used for both designs (helical and straight), i.e., the same volume. The length of the HF increases with the number of helical turns. Therefore, the most important performance indicator for a new design is to increase the module’s productivity, even if at the cost of a reduction in the flux in some cases. In comparison to using several straight hollow fibers, the use of a helical hollow fiber arrangement increases the packing density while maintaining the fibers in an orderly shape.

The current model can be extended to include correlations and models that describe different operational challenges, such as membrane pore wetting and scaling [37,38,39]. However, the comparisons between the typically used straight HF design and the proposed helical HF design were based on the same assumptions that ignored such operational conditions. Therefore, the final study outcomes can be justified in determining the best design on the same basis. Positively, the new design with helical HF modules is supposed to perform better than the straight hollow one regarding less scaling and fouling [35]. This also reduces the main cause of membrane pore wetting [40]. The reasons of membrane pore wetting include many factors that are independent of the membrane helical or straight form, such as pretreatment processes, membrane materials, etc.

In this study, the expected pressure drop per unit length and flux is depicted in Figure 10, and a compromise between the increased flux and pressure drop has been investigated to select a suitable feed velocity. Overall, the use of helical turns significantly enhanced the productivity and thermal performance of the module, and this will cost additional pumping power that needs to be considered in future economic investigations. Based on the initial estimation for a single helical HF unit comprising 1000 hollow fibers, each with 50 turns, the unit can produce 611 L/day compared to 418 L/day produced from a corresponding straight HF unit, while the daily energy consumption to pump the feed into the HF passages increases to 0.811 kWh instead of 0.406 kWh, respectively.

## 6. Conclusions

This study presents a novel configuration of HF membranes using helical turns to improve the productivity and thermal performance of the HF-WGMD modules. To numerically simulate all transport phenomena related to the distillation process in the proposed HF-WGMD module with minimal reliance on many assumptions, a 3D fully coupled CFD model is developed and validated. Single and double helical HF membrane designs with various numbers of turns are examined and contrasted with the conventional modules of single and double straight HF membrane designs, under various operational conditions. The primary conclusions are as follows:

An optimal distillate flux is consistently achieved with a helical turn count of 20. Increasing the number of turns beyond 20 results in larger thermal loads within the water gap, which adversely affects the flux generated. The greatest improvement of 11.4% is observed when comparing the flux at 20 helical turns with the reference module of a straight HF membrane at a 70 °C feed temperature.

The maximum specific productivity of 25.3 m_w_^3^/(m_du_^3^·day) is obtained from 50 turns in the case of single helical modules at a feed temperature of 70 °C, which represents a 46.2% improvement over the typical module of a single straight HF membrane. When using a double helical design under the same circumstances, this maximum productivity can be enhanced once more by 78% to be 45 m_w_^3^/(m_du_^3^·day).

With 50 single helical turns and a feed temperature of 70 °C, the best specific thermal energy consumption of 3.9 kWh/kg_w_ is achieved, providing a positive reduction in the energy consumption of 35% from the reference straight fiber case.

The system’s gain output ratio is increased by 117.6% when three stages of single helical modules are used in a series as opposed to one stage, while the specific thermal energy consumption is favorably reduced by 53.8%.

## Figures and Tables

**Table 1:** Geometrical characteristics and operational conditions of the simulated HF-WGMD unit.

Parameter	Symbol	Value	Unit
HF membrane inner diameter	$d_{mi}$	0.8	mm
HF membrane outer diameter	$d_{mo}$	1.16	mm
Cooling tube inner diameter	$d_{ti}$	5	mm
Cooling tube outer diameter	$d_{ti}$	5.56	mm
Cooling tube spacing	$P_{t}$	6.95	mm
Module effective length	L	500	mm
Feed inlet salinity	-	35,000	ppm
Water gap salinity	-	0.0	ppm
Coolant inlet temperature	$T_{ci}$	20	°C
Feed water thermal conductivity	$k_{f}$	0.64	W/m K
Membrane thermal conductivity	$k_{m}$	0.07	W/m K
Cooling tube thermal conductivity	$k_{t}$	0.445	W/m K
Membrane porosity	ε	82	%
Membrane pore tortuosity	τ	1.698	-
Membrane pore diameter	$d_{p}$	0.16	μm
Water vapor molar mass	$M_{w}$	18	g/mol
Salt molar mass	$M_{NaCl}$	58.4	g/mol

**Table 2:** The external boundary conditions of the simulated desalination unit.

Domain	Position	Boundary Conditions		
		Mass	Momentum	Energy
Feed channel	y=0	$c_{f}=c_{fi}$	$\overset{⃑}{u_{f}}=(0,U_{fi},0)$	$T_{f}=T_{fi}$
	y=L	$\frac{\partial c_{f}}{\partial y}=0$	$p_{f}=p_{atm}$	$\frac{\partial T_{f}}{\partial y}=0$
HF membrane	y=0	$\frac{\partial c_{m}}{\partial y}=0$	-	$\frac{\partial T_{m}}{\partial y}=0$
	y=L	$\frac{\partial c_{m}}{\partial y}=0$	-	$\frac{\partial T_{m}}{\partial y}=0$
Water gap	y=0	-	-	$\frac{\partial T_{g}}{\partial y}=0$
	y=L	-	-	$\frac{\partial T_{g}}{\partial y}=0$
Cooling tube	y=0	-	-	$\frac{\partial T_{t}}{\partial y}=0$
	y=L	-	-	$\frac{\partial T_{t}}{\partial y}=0$
Cooling channel	$x=0\;and\;x=P_{t}$	-	$\frac{\partial \overset{⃑}{u_{c}}}{\partial x}=\overset{⃑}{0}$	$\frac{\partial T_{c}}{\partial x}=0$
	$z=0\;and\;z=P_{t}$	-	$\frac{\partial \overset{⃑}{u_{c}}}{\partial z}=\overset{⃑}{0}$	$\frac{\partial T_{c}}{\partial z}=0$
	y=0	-	$p_{c}=p_{atm}$	$\frac{\partial T_{c}}{\partial y}=0$
	y=L	-	$\overset{⃑}{u_{c}}=(0,-U_{ci},0)$	$T_{c}=T_{ci}$

**Table 3:** Grid independence study on a case of single straight fiber with $T_{fi}=70\;{}^{\circ}C$ and $U_{fi}=0.81\;m/s$.

	Number of Grid Elements	Water Flux (L/(m^2^ h))	%Variation in Water Flux	Feed Outlet Temperature (°C)	% Variation in Feed Outlet Temperature
Grid 1	1,203,853	8.83	-	59.131	-
Grid 2	2,411,325	9.05	2.492	59.349	0.369
Grid 3	5,168,202	9	−0.552	59.438	0.15

**Table 4:** Operational and geometrical parameters of experimental modules used in CFD model validation.

Reference	Experimental Module	Number of Fibers per Tube	Module Length (mm)	Feed Inlet Temperature (°C)	Feed Inlet Velocity (m/s)
[28]	Module 1	1	350	40, 50, 60, and 70	0.69
	Module 2	2			
	Module 3	3			
[14]	Variable feed inlet temperatures	1	425		0.81
	Variable feed inlet velocities			70	0.28, 0.4, 0.53, 0.69, and 0.81

**Table 5:** Salinity and temperature at feed–membrane interface at fiber middle length (y=250 mm) of desalination unit with single straight fiber at different feed inlet velocities.

	$\mathrm{Feed}\;\mathrm{Inlet}\;\mathrm{Velocity}\;(U_{fi})$ (m/s)				
	0.29	0.58	0.87	1.16	1.45
Salinity (ppm)	65,401	62,941	61,552	60,269	58,945
Temperature (°C)	51.9	59	61.8	63.3	64.2

**Table 6:** Water gap average temperature at different cross sections along desalination unit with single and double straight fibers at different coolant inlet velocities.

		y = 125 mm	y = 250 mm	y = 375 mm
		WG Average Temperature (°C)		
$U_{ci}=0.0031\;m/s$	Single	61.3	56.4	48.2
	Double	67.5	64.7	58.6
$U_{ci}=0.0125\;m/s$	Single	44.6	41.6	37.7
	Double	55.2	50.6	46.3
$U_{ci}=0.05\;m/s$	Single	36.1	35.2	33.5
	Double	44.6	41.4	39.7
$U_{ci}=0.21\;m/s$	Single	33.4	33.2	32.2
	Double	40.4	38.1	37.5

**Table 7:** Water gap average temperatures with single and double fibers.

	WG Average Temperature (°C)					
	Straight	10 Turns	20 Turns	30 Turns	40 Turns	50 Turns
Single	31.4	30	30.5	31.2	32	32.9
Double	38.2	38.4	39.2	40.4	41.5	42.9

**Table 8:** Average water vapor concentration differences across the HF membrane with single and double fibers.

	Average Concentration Difference (mol/m^3^)					
	Straight	10 Turns	20 Turns	30 Turns	40 Turns	50 Turns
Single	4.116	4.435	4.51	4.439	4.293	4.206
Double	3.792	4.069	4.116	4.009	3.837	3.709

**Table 9:** Water vapor average concentration with 50 turns of double helical fibers.

	Feed Inlet Temperature (°C)			
	40	50	60	70
Feed–membrane interface	2.445	3.765	5.612	8.122
Membrane–WG interface	1.682	2.28	3.147	4.413
Concentration difference	0.763	1.485	2.465	3.709

**Figure A1:**
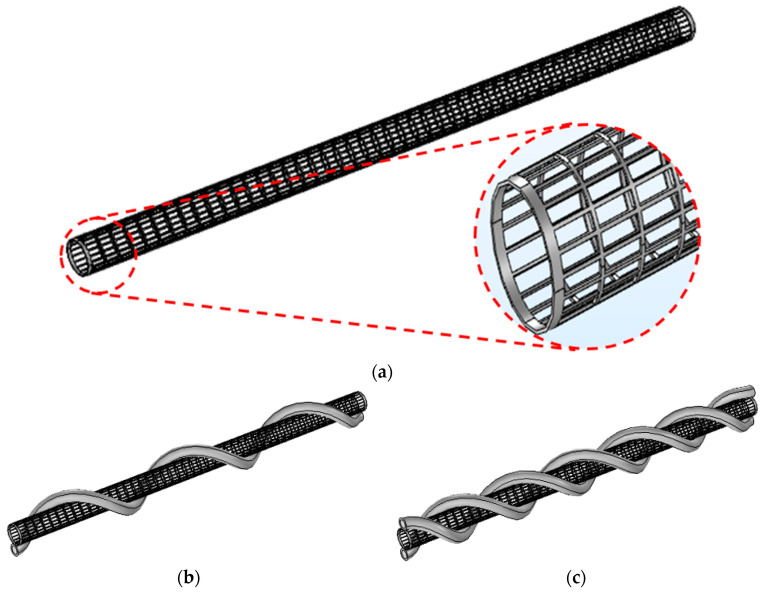
Schematic diagrams of (**a**) perforated support tube, (**b**) single helical fiber supported by the perforated tube, and (**c**) double helical fibers supported by the perforated tube.

## Data Availability

Not applicable.

## Nomenclature

$a_{w}$	Water activity coefficient
$C_{p}$	Specific heat at constant pressure (J/kg K)
c	Concentration (mol/m^3^)
D	Diffusion coefficient (m^2^/s)
$D_{Kn}$	Knudsen diffusion coefficient (m^2^/s)
$D_{Or}$	Ordinary molecular diffusion coefficient (m^2^/s)
d	Diameter (m)
$d_{p}$	Membrane pore diameter (m)
G	Diffusive flux (kg/(m^2^ s))
GOR	Gain output ratio
$h_{fg}$	Latent heat of vaporization (J/kg)
J	Flux (L/(m^2^ h))
Kn	Knudsen number
k	Thermal conductivity (W/m K)
L	Module effective length (m)
M	Molecular mass (g/mol)
p	Pressure (Pa)
$P_{t}$	Cooling tube spacing (m)
ppm	Concentration in parts per millions
q	Heat flux (W/m^2^)
$\bar{R}$	Universal gas constant (J/(mol K))
Re	$\frac{\rho U_{fi}d_{mi}}{\mu }$
SP	Specific productivity (m_w_^3^/(m_du_^3^ day))
STEC	Specific thermal energy consumption (kWh/kg_w_)
T	Temperature (°C)
TER	Thermal energy recovered (%)
*U*	Flow stream mean velocity
$\overset{⃑}{u}$	Flow velocity field vector (m/s)
v	Specific volume (m^3^/kg)
W	Water vapor content (kg_v_/kg_a_)
$x_{NaCl}$	Salt mole fraction
$x_{w}$	Water mole fraction
Subscripts	
a	Air
atm	Atmospheric
c	Coolant channel
du	Desalination unit
f	Feed channel
g	Water gap
i	Inlet
m	Membrane
NaCl	NaCl salt
o	Outlet
sat	Saturation
t	Cooling tube
v	Vapor
w	Water
Greek symbols	
ε	Membrane porosity
μ	Fluid dynamic viscosity (Pa s)
ρ	Fluid density (kg/m^3^)
σ	Molecule collision diameter (m)
τ	Membrane pore tortuosity

## Appendix A

### Appendix A.1. Proposal of Helical Fibers’ Support Tube

Figure A1 shows the perforated support tube that can be used to facilitate applying the proposed design for a single or double helical HF membrane with a minimal impact on the system’s performance.

### Appendix A.2. Mathematical Model

(1) Water–salt diffusion coefficient

The water–salt mutual diffusion coefficient $D_{w}$ is calculated using the Wilke–Chang equation [41]:

$$D_{w}=\frac{7.4\times 10^{-8}{\left(2.6M_{f}\right)}^{1/2}T_{f}}{\mu_{f}{V_{w}}^{0.6}}$$

where $M_{f}$ is the feed solution molar mass (solvent) in g/mol, $T_{f}$ is the local feed temperature in K, $\mu_{f}$ is the feed dynamic viscosity in cP, and $V_{w}$ is the water molar volume in cm^3^/mol.

(2) HF membrane diffusion coefficient

The diffusion of water vapor molecules through the hydrophobic membrane pores can be modeled using one or more diffusion mechanisms such as ordinary molecular diffusion, Knudsen diffusion, and Poiseuille flow. In order to determine the effective diffusion mechanisms in the current membrane model, the Knudsen number (Kn) must be calculated as follows [42]:

$$Kn=\frac{{Ɩ}_{a-w}}{d_{p}}$$

where ${Ɩ}_{a-w}$ is the mean free path of the water vapor molecule and $d_{p}$ is the membrane pore diameter, and ${Ɩ}_{a-w}$ can be estimated as follows:

$${Ɩ}_{a-w}=\frac{K_{B\;}T_{m,avg}}{\;p\;\pi \;{\left[\left(\sigma_{a}+\sigma_{w}\right)/2\right]}^{2}}\times \frac{1}{{\left(1+\left(M_{w}/M_{a}\right)\right)}^{1/2}}$$

where $K_{B\;}$ is the Boltzmann constant, $T_{m,avg}$ is the HF membrane average temperature, p is the membrane pore pressure, $\sigma_{a}$ and $\sigma_{w}$ are the air and water molecule collision diameters, respectively ($\sigma_{a}=3.71\;\dot{A}\;and\;\sigma_{w}=2.64\;\dot{A}$ [43]), and $M_{w}$ and $M_{a}$ are the water and air molecular masses, respectively ($M_{a}=29\;g/mol\;and\;M_{w}=18\;g/mol)$.

Therefore, for a membrane mean temperature range of $20\;{}^{\circ}C<T_{m}<70\;{}^{\circ}C$ and a membrane pore diameter of 0.16 μm, the calculated Knudsen number is in range of 0.1<Kn<10, which is considered as the transition region of diffusion. So, the combined ordinary molecular diffusion and Knudsen diffusion must be used to model the diffusion coefficient for vapor diffusion through the membrane pore, while the Poiseuille flow can be neglected due to the neglected pressure gradient across membrane pores. The membrane diffusion coefficient is calculated as follows:

$$D_{m}=\frac{\varepsilon }{\tau }\;D_{or-Kn}$$

where ε is the HF membrane porosity, and τ is the HF membrane pore tortuosity and can be calculated as follows [44]:

$$\tau =\frac{{\left(2-\varepsilon \right)}^{2}}{\varepsilon }$$

where $D_{or-Kn}$ is the combined ordinary molecular diffusion, and the Knudsen diffusion coefficient is calculated as follows:

$$D_{or-Kn}={\left(\frac{1}{D_{or}}+\frac{1}{D_{Kn}}\right)}^{-1}$$

where $D_{or}$ and $D_{Kn}$ are the ordinary molecular diffusion and Knudsen diffusion coefficients, respectively, and can be calculated as follows [42]:

$$D_{or}=1.97\times 10^{-5}{\left(\frac{T_{m}}{256}\right)}^{1.685}$$

$$D_{Kn}=\frac{d_{p}}{3}\times {\left(\frac{8\bar{R}T_{m}}{\pi M_{w}}\right)}^{\frac{1}{2}}$$

where $T_{m}$ is the HF membrane’s local temperature, $d_{p}$ is the membrane pore diameter, $\bar{R}$ is the universal gas constant, and $M_{w}$ is the water molecular mass.

(3) HF membrane thermal conductivity

The membrane overall thermal conductivity can be calculated using the following equation [45]:

$$k_{m}=\varepsilon k_{v}+\left(1-\varepsilon \right)k_{s}$$

where ε is the porosity of the HF membrane, $k_{s}$ is the thermal conductivity of the Polyvinylidene Fluoride HF membrane, and $k_{v}$ is the water vapor thermal conductivity and can be calculated using the method by Chen et al. [46] as follows:

$$k_{v}=0.0144-2.16\times 10^{-5}\;T_{m}+1.32\times 10^{-7}{T_{m}}^{2}$$

where $T_{m}$ is the HF membrane local temperature in K.

(4) Heat source and heat sink boundary conditions

The heat energy absorbed from the feed water by the water vapor due to evaporation at the feed–membrane (hot) interface and the heat energy expelled to the water gap due to condensation at the membrane–water gap (cold) interface are modeled using a heat sink and heat source boundary conditions at the hot and cold membrane interfaces, respectively. The amount of heat energy absorbed or expelled is calculated as follows:

$$q=G{\;h}_{fg}$$

$${h_{fg}=24.49-2.2T}_{m}$$

where q is the amount of heat flux absorbed from the feed water or expelled to the water gap, G is the diffusive vapor mass flux through the HF membrane layer, $h_{fg}$ is the latent heat vaporization or condensation, and $T_{m}$ is the membrane local temperature at the feed–membrane or membrane–water gap interfaces.

(5) Membrane interfaces’ boundary conditions

The air inside the HF membrane pores at both membrane interfaces can be considered saturated due to the direct evaporation and condensation processes that occur on both membrane sides. So, the vapor partial pressures at the membrane’s hot and cold interfaces can be calculated from the saturation pressures using Antoine equations, as follows [47]:

$$P_{sat,f-m}=x_{w}a_{w}\times 133.416{\times 10}^{8.10765-\frac{1750.286}{T_{m}+235}}$$

$$P_{sat,m-g}=133.416{\times 10}^{8.10765-\frac{1750.286}{T_{m}+235}}$$

where $P_{sat,f-m}$ is the saline water saturation pressure at the feed–membrane interface, $P_{sat,m-g}$ is the distillate water saturation pressure at the membrane–water gap interface, $x_{w}$ is the water molar fraction of the feed saline water, and $a_{w}$ is the water activity coefficient, and both can be calculated as follows [46]:

$$x_{w}=1-x_{NaCl}$$

$$a_{w}=1-0.5x_{NaCl}-10x_{NaCl}^{2}$$

where $x_{NaCl}$ is the local salt molar fraction of feed saline water.

Therefore, the water vapor concentrations at both membrane interfaces can be calculated using the saturation pressures as follows:

$$c_{sat}=\frac{W}{v_{a}M_{w}}$$

$$W=0.621945\frac{p_{sat}}{p_{atm}-p_{sat}}$$

$$v_{a}=0.28704{\;T}_{m}\frac{1+1.607858\;W}{10^{-3}\;p_{atm}}$$

where $c_{sat}$ is the saturation vapor concentration at both membrane interfaces, W is the vapor mass content, $v_{a}$ is the saturated air specific volume, and $p_{atm}$ is the atmospheric pressure.
